# Uracil-Containing DNA in *Drosophila*: Stability, Stage-Specific Accumulation, and Developmental Involvement

**DOI:** 10.1371/journal.pgen.1002738

**Published:** 2012-06-07

**Authors:** Villő Muha, András Horváth, Angéla Békési, Mária Pukáncsik, Barbara Hodoscsek, Gábor Merényi, Gergely Róna, Júlia Batki, István Kiss, Ferenc Jankovics, Péter Vilmos, Miklós Erdélyi, Beáta G. Vértessy

**Affiliations:** 1 Institute of Enzymology, Research Center for Natural Sciences, Hungarian Academy of Science, Budapest, Hungary; 2 Institute of Genetics, Biological Research Centre (BRC), Hungarian Academy of Science, Szeged, Hungary; 3 Department of Applied Biotechnology and Food Sciences, University of Technology and Economics, Budapest, Hungary; Stowers Institute for Medical Research, United States of America

## Abstract

Base-excision repair and control of nucleotide pools safe-guard against permanent uracil accumulation in DNA relying on two key enzymes: uracil–DNA glycosylase and dUTPase. Lack of the major uracil–DNA glycosylase UNG gene from the fruit fly genome and dUTPase from fruit fly larvae prompted the hypotheses that i) uracil may accumulate in *Drosophila* genomic DNA where it may be well tolerated, and ii) this accumulation may affect development. Here we show that i) *Drosophila melanogaster* tolerates high levels of uracil in DNA; ii) such DNA is correctly interpreted in cell culture and embryo; and iii) under physiological spatio-temporal control, DNA from fruit fly larvae, pupae, and imago contain greatly elevated levels of uracil (200–2,000 uracil/million bases, quantified using a novel real-time PCR–based assay). Uracil is accumulated in genomic DNA of larval tissues during larval development, whereas DNA from imaginal tissues contains much less uracil. Upon pupation and metamorphosis, uracil content in DNA is significantly decreased. We propose that the observed developmental pattern of uracil–DNA is due to the lack of the key repair enzyme UNG from the *Drosophila* genome together with down-regulation of dUTPase in larval tissues. In agreement, we show that dUTPase silencing increases the uracil content in DNA of imaginal tissues and induces strong lethality at the early pupal stages, indicating that tolerance of highly uracil-substituted DNA is also stage-specific. Silencing of dUTPase perturbs the physiological pattern of uracil–DNA accumulation in *Drosophila* and leads to a strongly lethal phenotype in early pupal stages. These findings suggest a novel role of uracil-containing DNA in *Drosophila* development and metamorphosis and present a novel example for developmental effects of dUTPase silencing in multicellular eukaryotes. Importantly, we also show lack of the UNG gene in all available genomes of other Holometabola insects, indicating a potentially general tolerance and developmental role of uracil–DNA in this evolutionary clade.

## Author Summary

The usual paradigm confines “normal” DNA of living cells to a well-defined restricted chemical space populated with only four bases (adenine, thymine, guanine, and cytosine) and some of their methylated derivatives (e.g. 5′-methyl-cytosine). Uracil is not considered to be a “normal” DNA base, except in several bacteriophages. On the contrary, uracil is generally considered to be an error in DNA. We show that *Drosophila* cells interpret uracil-substituted DNA as normal DNA, due to lack of two repair enzymes. Importantly, this unusual trait is under developmental control and applies only for animals before pupation. Metamorphosis is drastically perturbed by silencing of dUTPase, responsible for keeping uracil out of DNA. Our results argue that in *Drosophila*, and perhaps in other Holometabola insects as well, uracil–DNA plays a dedicated physiological role.

## Introduction

In wild-type organisms, notably excepting some rare bacteriophages with thymineless DNA genomes [1], [2], uracil in DNA is thought to occur only transiently and at very low frequency (<20/million bases) as a damage product [3], [4]. Efficient base-excision DNA repair together with fine-tuned control of nucleotide pools safe-guard against uracil in DNA relying on two key enzymes: uracil–DNA glycosylase (UDG) [5] and dUTPase [6].

UDG deficiency, in combination with thymidylate synthase inhibition or depleted dUTPase activity, was reported to lead into notable uracil accumulation in DNA. However, deficiency in uracil–DNA glycosylase on its own contributes only slightly to the genomic uracil content [7]–[10]. Since dUTPase deficiency or silencing can be rescued by depleted activity of UDG [11]–[14], it can be argued that UDG is a major factor that renders uracil–DNA, formed in absence of dUTPase, intolerable for cells. Uracil–DNA glycosylases represent a superfamily that involves enzymes with specialized functions. Among the superfamily members, UNG is reported to be the most abundant one that also possesses the highest activity in removing uracils from any context of both single-stranded and double-stranded DNA [5]. SMUG has similar attributes to UNG but prefers uracil-containing ssDNA as substrate [15]. MBD4 and TDG recognize mismatched uracil or thymine bases that base-pair with guanine [16]. The latter enzyme is known to function within CpG islands, where thymine is formed after spontaneous methyl-cytosine deamination [17].

Through hydrolyzing dUTP to dUMP and pyrophosphate, dUTPase serves a dual role in cell physiology: it produces a precursor for thymidylate synthesis, and removes dUTP from the deoxynucleotide pool. Eukaryotic and bacterial DNA polymerases incorporate deoxyuridine into DNA with a rate that depends on the cellular dUTP concentration [6], [10]. Therefore dUTPase has a significant indirect impact in prevention of uracil incorporation into DNA. Lack of dUTPase is supposed to induce thymine to uracil substitution that does not alter the genetic code; however UDG still removes this kind of base modification supposedly resulting in genome instability. This process may serve as an explanation for overall lethality of dUTPase deficiency [6].

Here, we propose that a condition similar to simultaneous deficiency in both dUTPase and UDG is present in *Drosophila* larvae. The *Drosophila* genome does not encode the major uracil–DNA glycosylase UNG [18], therefore uracil–DNA tolerance may be expected. *Drosophila* larvae contain both proliferating primordial tissues of imago and differentiated tissues that degrade during metamorphosis. Our previous study reported *Drosophila* dUTPase [19], [20] expression only in the first one of the two kinds of tissues [21], [22]. We wished to investigate if the stage- and tissue-specific expression of dUTPase is translated into differences in the uracil content of genomic DNA and whether this putative pattern has developmental significance.

We now report high resistance of *Drosophila* cell lines to fluorodeoxyuridine (FdUR) that induced high uracil–DNA levels. We show that in addition to tolerance of uracil-substituted DNA, *Drosophila* cells correctly interpret this unusual DNA both *in vitro* and *in vivo*. Moreover, in wild type *Drosophila* we observe stage- and tissue-specific changes in uracil-content of DNA that are inversely correlated to dUTPase expression and cellular fate. We propose that the observed pattern is due to the lack of the *ung* gene from the *Drosophila* genome paralleled with developmental down-regulation of dUTPase in larval tissues. To check whether the absence of dUTPase may be causative for uracil accumulation in DNA, we show that silencing of dUTPase in larvae expands the uracil–DNA pattern to imaginal tissues, as well. Interestingly, dUTPase silencing results in abnormal DNA strand breaks, cell death and developmental arrest in early pupal stage. This may indicate that tolerance of highly uracil-containing DNA is also stage-specific, and other tolerance factors, in addition to the lack of UNG, may appear in this specific developmental phase. To our knowledge, our study is the first one that reports uracil–DNA appearance in absence of dUTPase in a wild type complex eukaryotic organism and demonstrates the developmental pattern of its tolerance and stability.

## Results/Discussion

**Figure 1:**
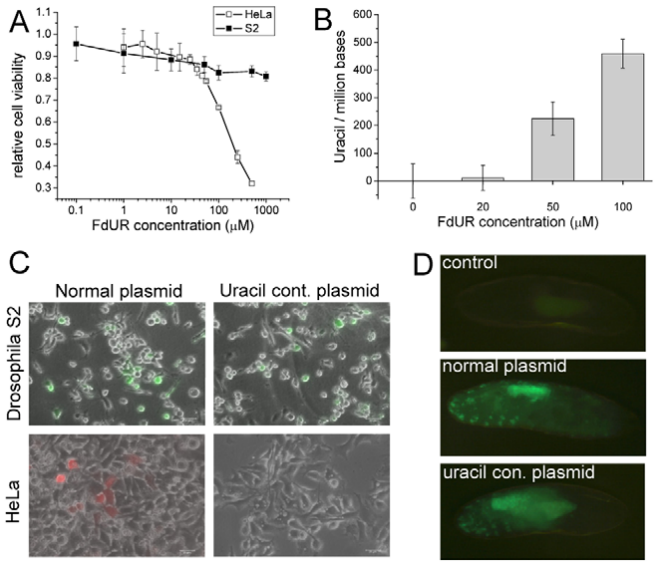
Tolerance and stability of uracil-containing DNA in *D. melanogaster*. (A) Dose-response curve upon FdUR treatment followed by Alamar Blue assay. (B) FdUR leads to uracil accumulation in DNA of *Drosophila* S2 cells. Data indicate that increased level of uracil is well-tolerated in *Drosophila*, but not in human cells. Data are presented as mean ± s.e.m. (C) *Drosophila* S2 (top panels) and human HeLa cells (bottom panels) were transfected with normal plasmid (left panels) or uracil-containing plasmid (right panels). Expression of YFP in *Drosophila* S2 cells or dsRedMonomer in HeLa cells indicates stability of the DNA. (D) Microinjection of uracil-plasmid into *Drosophila* embryo. Non-injected embryos served as control sample.

Since the *Drosophila* genome lacks UNG, we wished to test whether *Drosophila* cells show similar characteristics to UNG deficient organisms. The drug 5′-fluorodeoxyuridine (FdUR), frequently used as an inhibitor of thymidylate biosynthesis [23], [24], leads to perturbation of nucleotide levels and cell death. Loss of uracil–DNA glycosylase activity was found to lead to fluoropyrimidine resistance in *E. coli* [25], yeast [26] and *C. elegans* [27]. Deficient uracil–DNA glycosylase activity was also reported to be required for increased genomic uracil content after FdUR exposure in *E. coli* and mammalian cells [7]–[10]. Therefore, response to FdUR treatment may be an indicator for testing cellular uracil–DNA glycosylase activity. We observed that the *Drosophila* S2 cell line shows only small decrease in viability in the presence of 1 mM of FdUR, while HeLa cells, possessing the *ung* gene, show strong lethality at this drug concentration (Figure 1A). As a dose-dependent response to increasing concentrations of FdUR, uracil accumulation in genomic DNA of *Drosophila* S2 cells became highly elevated (up to approx. 450 uracil/million bases) (Figure 1B). Both the observed relatively high resistance for FdUR and the cellular response of genomic uracil incorporation may be explained by the fact that *Drosophila* cells lack significant uracil–DNA glycosylase activity.

We then asked if uracil-containing chemically unusual DNA may be tolerated and interpreted in *Drosophila* cells. Uracil–DNA specific cell response was provoked by transfecting cells with exogenous plasmid uracil–DNA. The expression signal of the fluorescent reporters (eYFP or dsRedMonomer) encoded by plasmids produced in wild type (normal plasmid) or *dut-1, ung-1* (uracil-plasmid, approx. 5500 uracil/million bases [8], [10]) *E. coli* were followed. The gene span for the eYFP or DSRed-monomer reporters included the promoter (CMV immediate early or metallothionein), the reporter protein gene (DSRed-monomer or eYFP), and the SV40 polyadenylation signal. The total length of these gene spans comprises 1515 or 1299 nucleotides. If synthesized in the double mutant *dut-1*, *ung-1 E.coli* strain, the average number of uracil substitutions on a single strand within these gene spans corresponds to 8.3 and 7.1, respectively. Human (HeLa) cells, possessing UNG, showed appreciable fluorescent signal only when transformed with normal plasmid, indicating that uracil-containing plasmid was not interpreted probably due to its degradation. On the contrary, *Drosophila* S2 cells could express reporter genes encoded either on normal or uracil containing plasmid at comparable level (Figure 1C and Figure S1).

Having established that *Drosophila* cells in *in vitro* culture may tolerate and do correctly interpret uracil-containing DNA, we wished to assess the fruit fly physiological response to uracil–DNA at the organism level. Therefore similarly to the cell culture reporter-assay, uracil–DNA plasmid was introduced to *Drosophila* embryo. Upon microinjection of pP{Gal4VP16} expression vector (where the 1000 nt segment includes the P-element promoter, the Gal4-VP16 fusion gene and the hsp70 terminator that may have an average number of 5.5 uracil substitutions if produced in double mutant *E. coli*) into P{mw^+^UASeGFP} transgenic animals, the strong eGFP signal indicated the stability of exogenous DNA (Figure 1D). In pre-hatching embryos eGFP signal was detected from both normal and uracil-containing DNA with commeasurable intensities. In both cases the expression pattern of the reporter construct is similar and eGFP is most pronounced in the gut. These results can be explained only by assuming that the genetic information stored in uracil-containing DNA serves as a cognate code for transcription in *Drosophila* cells. Such ability of the fruitfly cells is most probably due to lack of UNG under which condition uracil–DNA does not get rapidly degraded.

**Figure 2:**
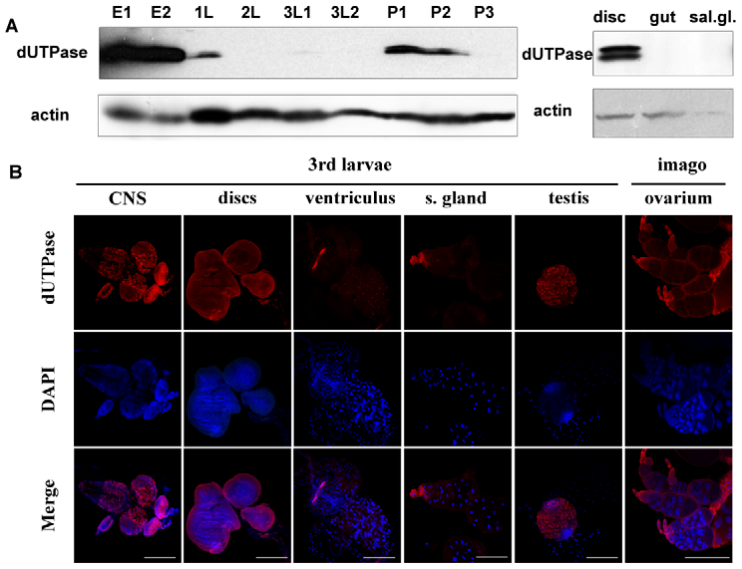
Stage- and tissue-specific distribution of dUTPase protein levels in *D. melanogaster*. Western blotting (A) and immunohistochemistry (B) was performed on selected developmental stages and tissues. Embryo 0–6 h (E1), embryo 0–24 h (E2), 1^st^ larvae (1L), 2^nd^ larvae (2L), early 3^rd^ larvae (3L1), wandering 3^rd^ larvae (3L2), pupae before head eversion (P1), pupae after head eversion (P2) and pupae 50–60 h after puparium formation (P3). For Western blotting, actin was used as loading control. Note that dUTPase protein levels are down-regulated during larval stages and expression is confined to specific tissues.

**Figure 3:**
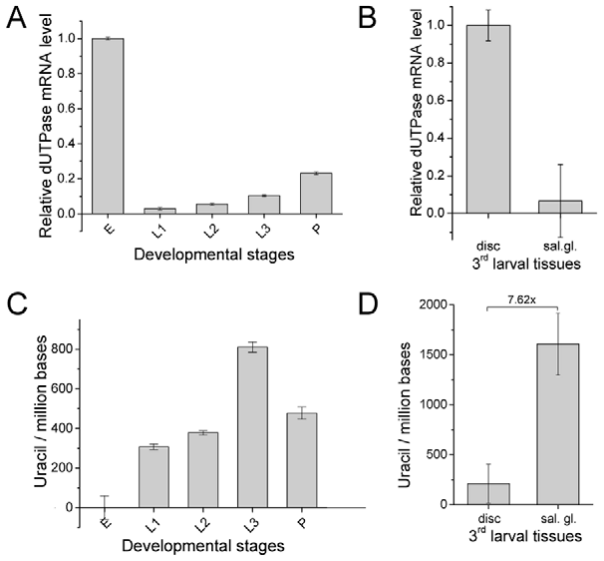
*D. melanogaster* genomic DNA uracil content inversely correlates with dUTPase expression. (A) Changes of dUTPase mRNA level throughout fruitfly development: embryo (E), 1^st^ larvae (L1), 2^nd^ larvae (L2), late 3^rd^ larvae (L3) and pupae (P). Note that dUTPase is down-regulated in larvae. (B) Comparison of dUTPase RNA level in the larval tissues salivary gland and imaginal tissue. Data are presented as mean of triplicates ± s.e.m. mRNA level was measured by RT-qPCR and dUTPase mRNA level was normalized to Rp49 mRNA level. (C) Uracil content of *D. melanogaster* genome in different developmental stages: embryo (E), 1^st^ larvae (L1), 2^nd^ larvae (L2), late 3^rd^ larvae (L3) and pupae (P). Embryonic sample was used as reference since it was shown to contain undetectable levels of uracil in DNA (cf. Figure S2). (D) Comparison of genomic uracil content in wild type imaginal disc and salivary gland of 3^rd^ larvae. Data are presented as mean ± s.e.m.

The other key factor responsible for keeping uracil out of DNA is the enzyme dUTPase, the importance of which is even more substantiated in *D. melanogaster* that lacks UNG. During development of *D. melanogaster*, high dUTPase protein levels can be observed only in embryonic stages (Figure 2A). As shown by Western blotting and immunohistochemistry, starting from late embryonic phase, dUTPase expression level is decreased drastically and remains low during postembryonic stages. Under normal physiological circumstances of larval development, dUTPase is predominantly expressed in imaginal discs, the central nervous system and in the testis (Figure 2) but not in most larval tissues, like salivary gland and gut. In the ventriculus and in the salivary gland, some cells are associated with dUTPase expression – these seem to correspond to the imaginal cells (Figure 2B). In the imago, dUTPase is present in the ovary of females. Cellular localization of dUTPase is usually nuclear [21], [28]–[31], however cytoplasmic occurrence is also evident in the nurse cells of mature follicles within ovaries, as described previously [21]. The present results are in agreement with our more limited earlier observations at the protein level [21]. We also quantified the dUTPase mRNA level by real-time PCR and found that protein and mRNA levels show similar tendencies during development (Figure 3A and 3B).

We were interested to learn whether coincidence of uracil–DNA tolerance and dUTPase down-regulation results in uracil accumulation in the genome of affected developmental stages and larval tissues. Determination of genomic uracil level was carried out by applying a recent quantitative real-time PCR-based method [10]. Data presented in Figure S2 and Figure 3C argue that DNA purified from embryo is relatively uracil-free, whereas in larval stages, uracil becomes much accumulated. The presence of uracil–DNA in larvae was further confirmed by using an independent method, the UNG-ARP assay (Figure S3) [8]. Uracil content also varies according to tissue type within the larvae: the salivary gland, a representative tissue of pupal degradation, accumulates high levels of uracil; while imaginal discs, which do not undergo abundant metamorphosis-coupled cell death, contain much less uracil (Figure 3D). Uracil level in larval tissues is comparable to that previously reported in the double mutant *dut1-1, ung-1 E. coli* strain [8], [10], amounting to several thousands of uracils per million bases. The above discussed pattern of uracil accumulation in different stages and tissues shows a clear negative correlation to the expression pattern of dUTPase (compare the respective developmental stages in Figure 2 and Figure 3).

**Figure 4:**
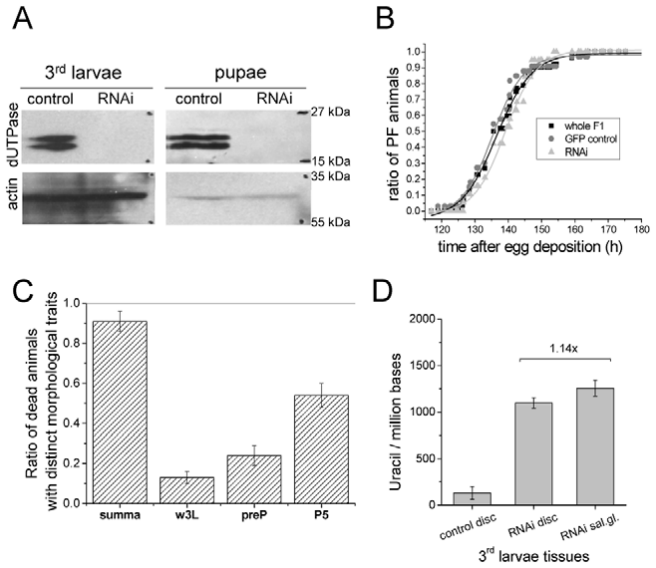
Silencing of dUTPase in *Drosophila* larvae and pupae. Western blots in (A) show that the protein level of dUTPase is under detection limit in silenced animals. Actin served as loading control. (B) Curves show the relative number of silenced and non-silenced animals that have undergone puparium formation at the given time point after egg deposition. Inflection points of the curves represent the mean time of puparium formation characteristic for the given population. dUTPase silencing did not perturb the time interval required for puparium formation. (C) Graph shows the number of counted dead animals relative to number of hatched curly winged control flies. Among these dead animals, three groups with distinct morphological traits characteristic for wandering larvae (w3L), prepupae (preP), and pupal stage P5 (P5) were identified and counted. (D) Genomic uracil content of dUTPase silenced and control tissues from 3^rd^ larvae.

**Figure 5:**
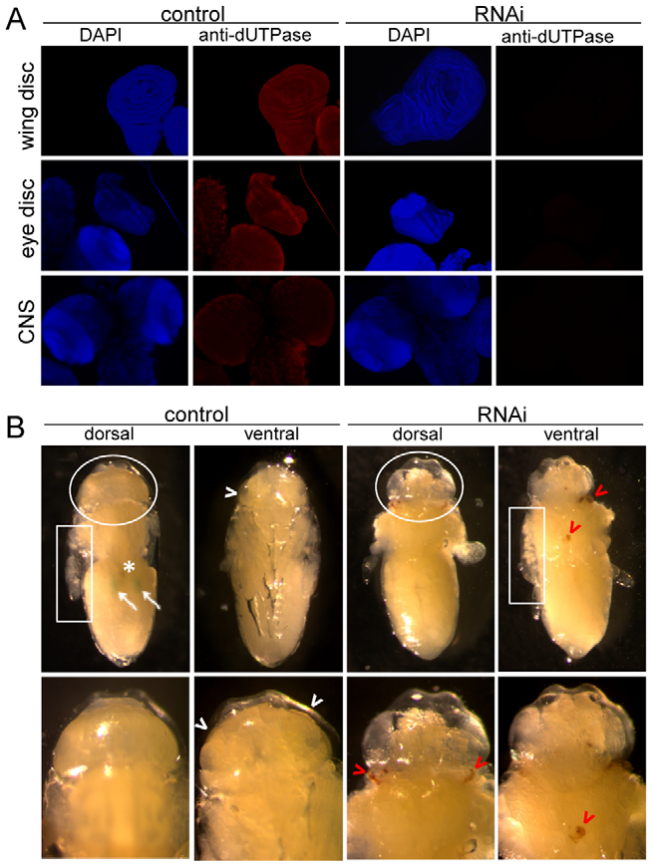
Morphological consequences of dUTPase silencing. In larvae (A) and pupae (B). (A) Immunohistochemistry of wing and eye discs, and brain of non-silenced (control) and silenced larvae for dUTPase (red) and DAPI staining for DNA (blue) demonstrate on one hand highly effective silencing; and on the other hand no observable morphological changes within these tissues. (B) Wild type pupae (control) in stage P6 (cf. Figure S5) and dUTPase silenced pupae at corresponding time after puparium formation in dorsal and ventral view are shown, after puparium removal. Wild type traits, Malpighian tubules (white arrows), Yellow Body (white asterix), developing adult eye (white arrowheads) are not observable on silenced animals. Instead, darkened (apoptotic/necrotic or melanized) tissues (red arrowheads) can be visualized on these pupae. Note the basically different inner texture of the everted discs (white boxes) and head sack (white circles).

In order to investigate if perturbation of the wild-type pattern of uracil levels in DNA may interfere with normal development, we aimed to silence dUTPase in transgenic *D. melanogaster* strains (Table S1). Efficient silencing could be successfully achieved in a setup resulting in well distinguishable F1 phenotypes: non-silenced animals were characterized by GFP expression and curly wings, whereas silenced animals had no markers [32] (Figure S4). Overall silencing resulted in efficiently decreased dUTPase protein levels in larvae and pupae (Figure 4A). RNAi silencing may not operate appropriately in early embryo due to maternal effects, but this stage is out of the scope of our present experiment with transgenic strains. We observed that dUTPase silencing did not perturb normal life and development of larvae. The silenced versus non-silenced larvae of F1 generation were selected based on GFP expression, and the time interval between the egg laying and puparium formation did not show any significant alteration (Figure 4B). Importantly, effective silencing of dUTPase in imaginal discs and larval brain did not cause any observable morphological changes in tissue morphology (Figure 5A). At pupal stage, however, dUTPase silencing induced 100% lethality, i.e. no silenced animals could develop into imago (observation is based on counting 2350 curly winged control imagos resulting from the first generation of crossings) (Figure 4C).

One possible proof for RNAi specificity is a rescue by the corresponding complementary DNA [33], [34]. Therefore, to test the specificity of dUTPase RNAi, the RNAi construct was co-expressed with a dUTPase transgene that led to the expression of dUTPase protein, as detected in the rescued animals (cf. method described in Text S1 and shown on Figure S4C and S4D). We observed a full rescue of the lethality caused by the RNAi confirming that the RNAi phenotype was due to a reduction of dUTPase level (Table S2). Rescued animals underwent development and metamorphosis just like the wild type animals. In the larval, prepupal and imago stages, rescued animals were carefully investigated for morphology and no distinct traits were observed.

In the detailed phenotype analysis of dUTPase-silenced animals, morphologic observations indicated serious adverse effects and developmental arrest at or before the pupal stage P5 [35] (Figure 5B, Figures S5 and S6). Upon removing the puparium of the silenced animal, defects were identified in the inner texture of everted discs, and head sack, as well as in the development of adult eye. These defects are permanent, and are not simply due to slow down of normal development. Moreover, 3–4 days after puparium formation, tissues showing morphological traits associated with the larval stage were identified within dissected samples (Figure S7 versus Figure S8). Noteworthy, the typical organ structures of the wild type adult (legs, wings, Malpighian tubules, Yellow Body, eyes etc.) never appeared in the silenced animals. Darkened tissues that may result from cell death, necrosis or histolysis were also observed e.g. in the prothoracic region and organs (Figure 5B and Figure S8) [36]. This result argued that although dUTPase is dispensable in larval tissues [21], the presence of the enzyme at the developmental stage of early puparium formation is essential for normal development during metamorphosis.

Tissue specific silencing in the dorsal wing surface of imaginal discs was also developed as described in Figure S9 (cf. [37]). The great majority of silenced animals developed curly wings, and a significant portion of them even showed blisters on the wings. Curling and blistering of the wings was suggested to result from abnormal cell death in dorsal wing surface [38], [39]. These results suggest that dUTPase silencing within a well-defined tissue segment may be specifically and exclusively associated with phenotypic effects in the very same tissue segment.

To analyze whether dUTPase silencing changed the distribution of uracil–DNA within different larval tissues, we assayed genomic uracil content of imaginal discs from both non-silenced and silenced larvae of F1 generation. As Figure 4D shows, imaginal discs from dUTPase silenced larvae accumulated a high amount of uracil in their genome (1097+/−55 uracil/million bases, to be compared with 131+/−69 uracil/million bases in the non-silenced animals) that approximated the genomic uracil content of salivary gland. Further increase in uracil–DNA content of salivary gland DNA was not observed in dUTPase silenced larvae, indicating that silencing was effective in tissues that normally express dUTPase but had no effect on uracil–DNA in tissues that normally do not express dUTPase. This result confirms that uracil appearance in DNA depends on dUTPase expression, thus dUTPase activity is causative in maintaining DNA with low levels of uracil.

**Figure 6:**
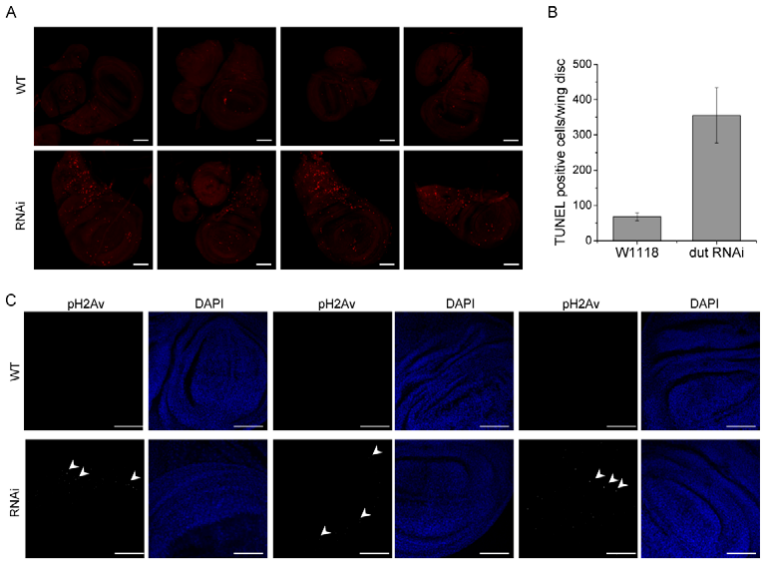
dUTPase silencing results in cell death and DNA strand breaks in larval imaginal discs. (A) Imaginal discs were isolated from wild type and dUTPase silenced wandering 3^rd^ larvae and stained for TUNEL assay (shown as red dots). Discs from silenced animals showed highly increased TUNEL staining. (B) TUNEL positive cell counts in imaginal discs from wild type and dUTPase silenced wandering 3^rd^ larvae. Error bars represent the standard error of mean. (C) Imaginal discs from wild type and dUTPase silenced 3^rd^ wandering larvae stained against phospho-H2Av foci (white dots, some of these are appointed by white arrowheads). dUTPase depleted discs showed several nuclei with phospho-H2Av foci indicating DNA damage. Scale bar represents 50 µm.

To analyze if the effects of dUTPase silencing may lead to DNA damage or cell death, we applied TUNEL and phospho-Histone H2Av assays [40], [41]. We observed that imaginal discs isolated from 3^rd^ stage wandering larva of dUTPase-silenced animals show strong enrichment in TUNEL positive cells (Figure 6A and 6B). TUNEL staining in the tissues indicate primarily cell death in the phase of DNA fragmentation. To address the question whether dUTPase depletion violates genome integrity we stained nuclei for phospho-Histone H2Av. H2Av histone modification by the ATR/ATM kinases indicates DNA double strand breaks (DSBs) in the proximity of the foci [41]. We observed numerous phospho-H2Av foci in dUTPase silenced wing imaginal discs, while no such foci were visible in the wild type (Figure 6C). These results suggest a potential correlation between dUTPase activity and DNA integrity. The increased level of DNA damage observed in our experiments in the dUTPase-silenced animals may result from excessive processing of uracil-containing DNA that concludes to DNA fragmentation.

Uracil–DNA measurements provided direct evidence that larval tissues of *D. melanogaster* that undergo developmental degradation accumulate uracil in genomic DNA. Up to date, presence of highly uracil-substituted genomic DNA within wild type organisms was reported only in some viruses; e.g. in bacteriophages [1], [2] and recently in HIV. To our knowledge, the present study may present a first example for naturally occurring permanent existence of uracil–DNA in a free-living complex eukaryotic organism (cf. Figure 3C and 3D). This condition requires developmental down-regulation of dUTPase and absence of UNG in the case of *D. melanogaster*. Involvement of dUTPase in determining genomic uracil content by regulating dUTP levels was confirmed by dUTPase silencing that resulted in appearance of uracil–DNA in imaginal disc tissues (cf. Figure 4D). Silencing of dUTPase resulted in developmental defects and DNA strand breaks (cf. Figure 5 and Figure 6). We also reported that uracil–DNA is tolerated and interpreted at least from embryonic to 3^rd^ larval stages.

The extraordinary situation of tolerance and stability of uracil–DNA may not be exclusively present in *D. melanogaster* as absence of *ung* is ubiquitous among Holometabola (Table 1). As uracil–DNA naturally occurs in larval tissues that are sentenced to death, we consider that uracil–DNA might be linked to metamorphosis and tissue degradation. Further investigations should be taken to describe the mechanism, its impact and its putative role.

**Table 1:** Occurrence of genes encoding dUTPase and UNG in different insects.

Insecta	Holometabola	Diptera		dUTPase	UNG
✓	✓	✓	*Drosophila melanogaster* (fruit fly)	+	−
✓	✓	✓	*Culexpipiens*(mosquito)	+	−
✓	✓	✓	*Aedesaegypti*(mosquito, yellow fever)	+	−*
✓	✓	✓	*Anopheles gambiae*(mosquito, malaria)	+	−
✓	✓		*Bombyxmori*(silkmoth)	+	−
✓	✓		*Triboliumcastaneum*(red flour beetle)	+	−
✓	✓		*Apismellifera*(honey bee)	+	−
✓	✓		*Nasoniavitripennis*(parasitoid wasp)	+	−
✓			*Acyrthosiphonpisum*(pea aphid)	+	−
✓			*Pediculushumanuscorporis*(body louse)	+	+

The gene for dUTPase is ubiquitous, but the gene of the major uracil–DNA glycosylase, ung is not encoded in the genome of Holometabola species.

*: In the genome of *Aedes aegypti* strain Liverpool, an unexpected ung sequence was found, showing very high (87%–94%) similarity to the ung gene of Comamonadaceae, a family of Proteobacteria, arguing for its bacterial origin.

Pupal lethality was observed in flies affected by mutations or silencing of purine biosynthesis enzymes [42], [43]. These animals showed apoptosis in developing imaginal primordium during metamorphosis that were also observable as darkened tissues. In these studies, deficient deoxynucleotide biosynthesis may have resulted in imbalanced dNTP levels and increased ratio of mismatches in DNA [44]. Overrepresented DNA modifications like cytosine methylation also result in pupal lethality [45], [46]. In this case increased methylation pattern of DNA inhibits transcriptional activity and cell cycle progression.

We can suggest two hypotheses to explain pupal intolerance of uracil–DNA. First, similarly to the case of hypermethylated DNA, uracil–DNA may show a decreased response and interaction with transcriptional regulators, activators or other morphogenetic factors required specifically during pupal metamorphosis. Second, one or more factor(s), functional only in the pupal stage, may process uracil–DNA resulting in genome instability and defects in cell cycle progression or cell death [47], [48]. Beyond UNG that is missing from *Drosophila*, other uracil–DNA glycosylases would be suspect for this role. In agreement with this suggestion, expression patterns of other uracil–DNA glycosylases (SMUG and Thd1) and AP endonucleases (Rrp1, RpS3 and RpLP0) indicate relative upregulation during metamorphosis after their lowest expression in larval stages according to microarray data (Table S3) [49]. Developmental downregulation of these base-excision DNA repair pathway (BER) enzymes may contribute to uracil–DNA appearance and tolerance in larval stages, and their upregulation may initiate uracil–DNA processing, destabilization or degradation. The pupal uracil–DNA intolerance pathways hypothesized above are intended to be analyzed in further experiments.

Taken together, we suggest three different factors as being responsible for the stage-specific elevated level of uracil in larval DNA: 1) lack of *ung* gene, 2) absence of dUTPase and 3) decreased levels of enzymes involved in uracil removal. We also conclude that dUTPase is essential for the full completion of the *Drosophila* life cycle, although its absence may be tolerated in the larval stages. Although fruit flies, and perhaps other Holometabola insects, are unique in possessing a developmental period when uracil–DNA can be tolerated, preservation and transmission of genetic information for several generations still requires dUTPase.

## Materials and Methods

### Uracil–DNA stability assays

For the uracil–DNA stability assays, pRm-eYFP, pDsRedMonomer-N1, pP{Gal4VP16} (kind gift from László Sipos) were amplified in *E.coli* K12 XL1Blue strain and CJ236 *dut-1, ung-1* strain (NEB). Plasmids were purified with Plasmid Midi Kit (QIAGEN). Uracil content of the plasmids was checked with UDG and AP endonuclease treatment followed by standard agarose gel electrophoresis [48].

#### Uracil-containing plasmid stability in cell culture

Transfection of pRm-eYFP-N-C* into *Drosophila* S2 cells was carried out in the presence of Cellfectine (Invitrogen) following the manufacturer's instructions and expression was induced from the metallothionein promoter at 25°C by addition of 700 µM CuSO_4_ and overnight incubation. pDsRedMonomer-N1 was transfected into HeLa cells by using Lipofectamine (Invitrogen). Samples were visualized with a Leica DMLS fluorescence microscope 48 hours after transfection.

#### Uracil-containing plasmid stability in *Drosophila* embryos

pP{Gal4VP16} plasmids were injected into 0–30 min pP{mw^+^UASeGFP} transgenic *Drosophila* embryos (kind gift of László Sipos). For each experiment, app. 40 dechorionated embryos were microinjected. They were aligned on a glass coverslip, dried for 30 min, than covered with 10S Voltalef oil before injection. Plasmid concentration was adjusted to 1 mg/ml, by dilution in standard injection buffer. GFP signal was detected in pre-hatching embryos, 22 h after injection. Embryos without injection served as a negative control.

### Cell viability assay

*Drosophila* Schneider 2 (S2) cells and human HeLa cells were cultured in 96 well plates at 5×10^4^cells/well or 2×10^3^ cells/well, respectively. The culture media used were Serum Free Insect Medium (Sigma, S3777) supplemented with 1% penicillin–streptomycin solution for S2 cells; and DMEM-F12 (Sigma, D8437) supplemented with 10% FBS and 1% penicillin–streptomycin for HeLa cells. FdUR (Sigma) was added at a final concentration range of 0.1–1000 µM. After 96 hours in culture, cell viability was quantified by Alamar Blue assay (BioSource). The experiment was repeated in triplicates.

### Quantitative measurement of the uracil content in DNA samples

In order to quantify uracil content of DNA, a real-time quantitative PCR-based assay was used [10]. Genomic DNA was isolated and digested with NheI. DNA fragments of 4–5 kb were purified from gel. Real-time PCR was performed on Mx3000P qPCR System (Agilent Technologies) using EvaGreen dye (Biotium) and PfuTurbo Hotstart DNA polymerase and PfuTurbo Cx Hotstart DNA polymerase (Stratagene). A segment with 963 base length defined by the primers (puBSd-Fw 5′-TCGGGATGACTTTTGGGTTCTG-3′ and puBSd985R 5′-CGCGGTTTAACACAGCGTCGG-3′) is amplified during the PCR reaction. Two-fold dilution series were prepared from DNA samples. Three or more parallel measurements were performed.

### dUTPase silencing by RNA interference and rescue of RNAi

UAS-IR stocks were obtained from Vienna *Drosophila* RNAi Centre (VDRC) [32]. Strain #21883 and #21884 was used for dUTPase silencing. Rescue was performed by co-expression of the RNAi construct with a dUTPase transgene. For details see Text S1, Tables S1 and S2, Figures S4 and S9.

### Immunohistochemistry and Western blot analysis

Western blot analysis of larval organs and stage specific dUTPase expression was performed as described previously [21]. For immunohistochemistry, applied primary antibodies were anti-dUTPase (1∶10000) or mouse anti-phospho-Histone H2A.X (Ser139) (Millipore) (1∶250). The latter was shown to recognize Drosophila phospho-Histone H2Av [50]. Tissues were fixed in 50% n-Heptane and 50% PEM-formaldehyde (100 mM PIPES, 1 mM MgCl_2_, 1 mM EGTA, 2.5% Tween-20, 4% PFA, pH = 6.9) for 30 minutes, with vigorous shacking at room temperature (RT). Samples were washed with inactivating buffer (50 mM TRIS, 150 mM NaCl, 0.5% Tween-20, pH = 7.4). Blocking was performed in the following: 5% goat serum, 1.5% BSA, 0.1% Tween-20, 1% Triton-X 100, 0,001% NaN_3_, in PBS, pH = 7.4 for 4 hours at RT. Tissues were incubated in primary antibody diluted in blocking buffer (1∶10000), at 4°C for 16 h. Samples were further washed with blocking buffer for 8 h at RT. Secondary antibody was applied to visualize dUTPase staining (Alexa 543 conjugated anti rabbit IgG, Molecular Probes) in blocking buffer (1∶1000) for 2 h, RT. DAPI was used for DNA staining. After further washing steps, samples were mounted in FluorSave (Calbiochem). Images were either acquired with a Zeiss LSCM 710 or a Leica DM IL LED Fluo microscope equipped with a Leica DFC345 FX monochrome camera.

### TUNEL assay

TUNEL assay was carried out as described in [40]. After careful dissection in PBS, imaginal discs were fixed (0.1M PIPES, pH = 6.9, 1 mM EDTA, 1% Triton X-100, 2 mM MgSO4, 1% formaldehyde) for 30 min at RT, washed for times with PBS buffer also containing 0.1% Triton X-100 (PBT) and two times with PBT 5X (PBS, 0.5% Triton X-100) (10 min each), and transferred into permeabilization solution (0.1M sodium citrate in PBT) for 30 min at 65°C. Discs were washed twice with PBT 5X, three times in PBT and incubated in reaction buffer (30 mM Tris-HCl, pH = 7.2, 140 mM sodium cacodylate, 1 mM cobalt chloride) for 30 min RT. Reaction was carried out in reaction buffer containing 0.2 unit/microL terminal deoxynucleotidyl transferase (NEB) and 5 microM Cy5-dUTP (GE Healthcare) for 1 hour RT. The reaction was stopped with PBT 5X and washed three times with PBT and finally with PBS. Nuclei were stained by Hoechst. Discs were imaged by laser scanning confocal microscopy (Zeiss LSCM 710). The number of TUNEL positive cells was determined by ImageJ (Rasband, W.S., ImageJ, National Institutes of Health, Bethesda, Maryland, USA, http://rsb.info.nih.gov/ij/, 1997–2004.)

## Supporting Information

Figure S1 Percentage of fluorescent cells upon transfection with normal (T pl.) or uracil-substituted plasmids (U pl.). (A) *Drosophila* S2 cells, (B) HeLa cells. The number of observed fluorescent cells is also presented within the bars together with the total number of scored cells (shown in brackets).
(PDF)

Figure S2 Genomic uracil content of embryo is under detection limit. Uracil content of *Drosophila* embryonic genome compared to that of DNA plasmid purified from wild-type *E. coli*. Both of the samples showed a value under the detection limit.
(PDF)

Figure S3 Ung-ARP assay. UNG-ARP assay shows presence of uracil–DNA in *Drosophila* larvae. For negative and positive controls, genomic DNA samples from XL1 Blue and CJ236 *ung-1, dut-1 E.coli* strains were used respectively. CJ236 *ung-1, dut-1 E.coli* strain produces DNA with high uracil content (approx. 5500 uracil/million bases [8], [10]).
(PDF)

Figure S4 Scheme of crossing for silencing of dUTPase in *Drosophila* larvae and pupae and for rescue of dUTPase RNAi. Crossing schemes are shown on panel A and C: Act-Gal4 means Gal4 gene coupled with actin 5C promoter that result in ubiquitous and constitutive expression of yeast transcription factor, Gal4 in transgenic *D. melanogaster* driving transcription of silencing element (IR) following the UAS promoter. F1 generation has two genotypes: Act-Gal4/UAS-IR animals express dsRNA for dUTPase silencing, and have no markers; in UAS-IR/CyO, GFP animals, the silencing element is not activate, curly wing (CyO) and GFP markers expressed. Silenced and non-silenced animals are distinguishable at larvae/pupae and imago stages on the basis of GFP (panel B) and CyO markers, respectively. Crossing scheme for silencing is shown on panel C: UAS-dUTPase-FLAG stands for the rescue construct. Two relevant categories of the F1 generation can be unambiguously distinguished based on the phenotype of the marker mutations of the CyO, SM6b, and TM3 balancer chromosomes. The TM3 phenotype marks the gene silenced progenies, while the rescued animals show noTM3 phenotype. Panel D shows Western blot for dUTPase in silenced versus rescued animals. Note the absence of dUTPase protein in silenced animals (silencing alleles 21883 and 21884), whereas the presence of dUTPase proteins in the rescued animals (rescuing alleles DMDUT20 and DMDUT29). Equivalent total protein loading was verified by developing the blot also against tubulin using anti-Tubulin (E7, provided by M. Klymkowsky; Developmental Studies Hybridoma Bank, University of Iowa, Iowa city, IA).
(PDF)

Figure S5 Summary of pupal developmental processes. Red arrow shows the stage P5 (around 12–14 h after puparium formation) until lethality due to dUTPase silencing appear.
(PDF)

Figure S6 Developmental arrest caused by dUTPase silencing in *Drosophila* pupae. Wild type (upper panels) and dUTPase silenced (bottom panels) pupae were compared in stages P4, P5–6, P6–7, and P9. Every panel shows four views of the same pupa: dorsal (upper two) and ventral (bottom two) with and without its puparium. Specific differences appear at or before P5: Malpighian tubules (arrows) and Yellow Body (asterices) never appears in dUTPase silenced pupae.
(PDF)

Figure S7 Wild type structures of pharate adults 3 days after puparium formation. Wild type pupa was dissected at stage P11 where adult organs have already developed (A). Dissected Malpighian tubules (arrows on B) and Yellow Body (asterices on B) of wild type pupa these organs have never identified within dUTPase silenced pupae.
(PDF)

Figure S8 Larval traits in dissected silenced pupae 3 days after puparium formation. Three days after puparium formation, dissected tissues of silenced pupae still preserve larval traits: testis is oval (A), foregut and gastric caeca show larval characteristics (B, D, asterices), Malpighian tubules (B, arrows) are thin characteristic for larval ones, and brain (C, white arrowhead) also preserves the basic structure of larval one. Darkened tissues may have resulted from necrosis, apoptosis or melanisation [36].
(PDF)

Figure S9 Scheme of crossing for silencing of dUTPase in the dorsal compartment of *Drosophila* wing imaginal discs. Crossing scheme is shown on panel (A): virgin females of the MS1096 Gal4 enhancer trap line expressing Gal4 preferentially in the dorsal compartment of the wing and carrying UAS-Dicer2 in homozygous form on the second chromosome (Bloomington stock No. 25706) were crossed to males carrying the Gal4 inducible silencing element (UAS-IR) in homozygous form on the second chromosome. The silencing element was activated by the MS1096 driver [37] in female progenies only while F1 males served as an internal negative control where no silencing occurred. Silenced females exhibited dorsally curled wing phenotype (panel B) often with blisters. The penetrance of the phenotype was around 85%. About 35% of the silenced female progeny also showed blistering wings (panel C). Male progenies had no wing phenotype. Panel D shows the expression pattern of the MS1096 driver in the dorsal compartment of the wing disc visualized by crossing MS1096 females to UAS-MoesinCherry [51] males (panel D) (red fluorescent staining in the wing disc). MoesinCherry overexpressing female progeny had no wing phenotype.
(PDF)

Table S1 Genomic position of UAS-IR constructs in dUTPase RNAi stocks.
(PDF)

Table S2 dUTPase transgene rescues the dUTPase RNAi phenotype. Table shows the results of the rescue crosses. UAS-IR/SM6b; UAS-dUTPase-FLAG/TM3 males were crossed to Act-Gal4/CyO females (Figure S4). Two UAS-IR (21883 and 21884) and two transgenic rescue lines (DMDUT20 and DMDUT29) were combined. Number of progenies of the relevant F1 categories is shown. Gene silencing was complete since no UAS-IR/Act-GAl4; TM3/+ adult progeny was observed. However, when the dUTPase transgene was present, rescued animals survived to adulthood.
(PDF)

Table S3 Uracil–DNA repair is perturbed in *Drosophila*. Microarray data available on FlyBase were used. Table shows mRNA level for genes involved in different DNA repair pathways, elements of uracil–DNA repair are highlighted on grey background. ↓ indicates mRNA level decrease, ↑ mRNA level increase, ≈ no stage-specific change. Note that the overall base excision repair is down-regulated during larval development, but other DNA repair processes are not.
(PDF)

Text S1 Supplementary information. Includes Supplementary Materials and Methods and Supplementary References.
(DOC)
