# Supplementary figures and images for: Uracil-Containing DNA in Drosophila: Stability, Stage-Specific Accumulation, and Developmental Involvement
Source: PLoS Genet. 2012 Jun 7;8(6):e1002738. doi: 10.1371/journal.pgen.1002738 (PMC3369950; doi:10.1371/journal.pgen.1002738)

**A**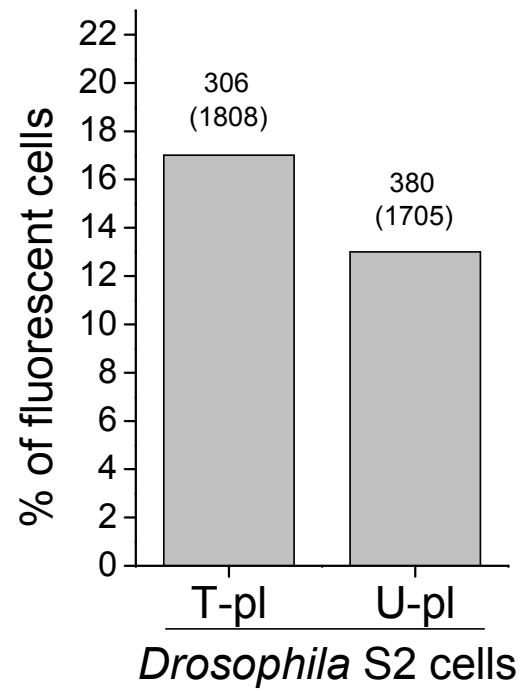**B**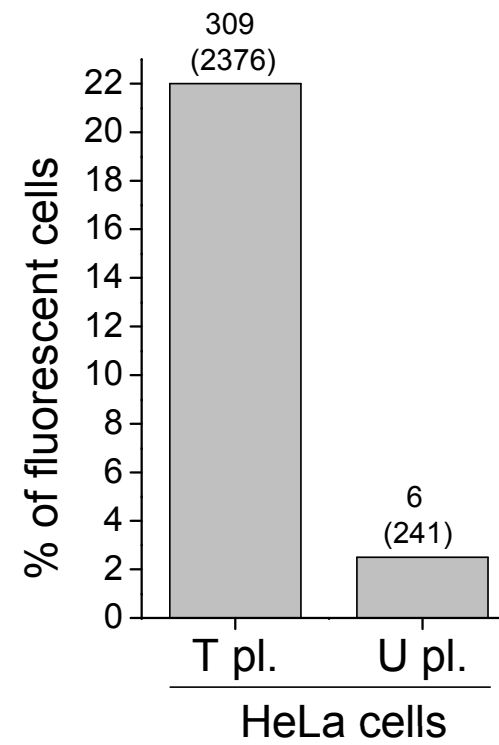

**Supplementary Figure S1**

Supplement: Figure S1 — Percentage of fluorescent cells upon transfection with normal (T pl.) or uracil-substituted plasmids (U pl.). (A) Drosophila S2 cells, (B) HeLa cells. The number of observed fluorescent cells is also presented within the bars together with the total number of scored cells (shown in brackets). (PDF) [file pgen.1002738.s001.pdf]

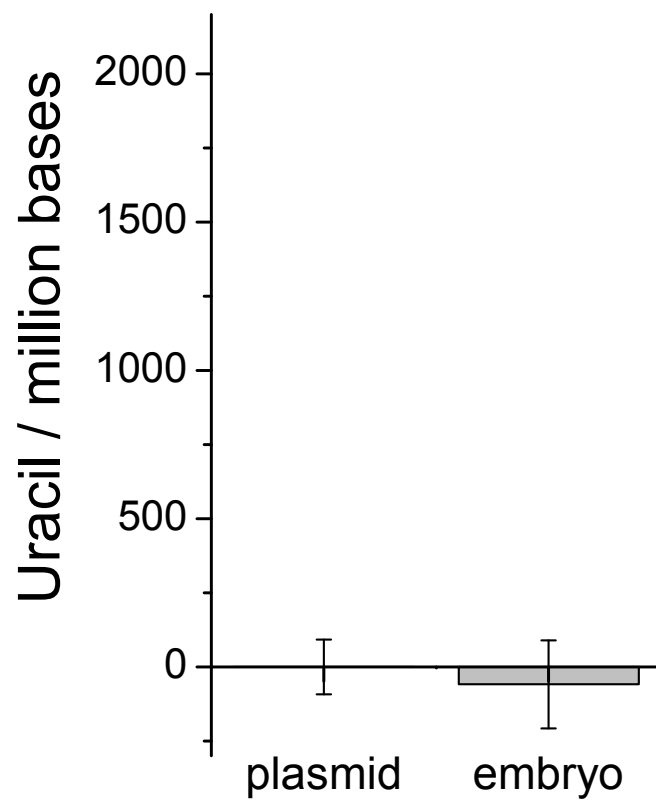

**Supplementary Figure S2**

Supplement: Figure S2 — Genomic uracil content of embryo is under detection limit. Uracil content of Drosophila embryonic genome compared to that of DNA plasmid purified from wild-type E. coli. Both of the samples showed a value under the detection limit. (PDF) [file pgen.1002738.s002.pdf]

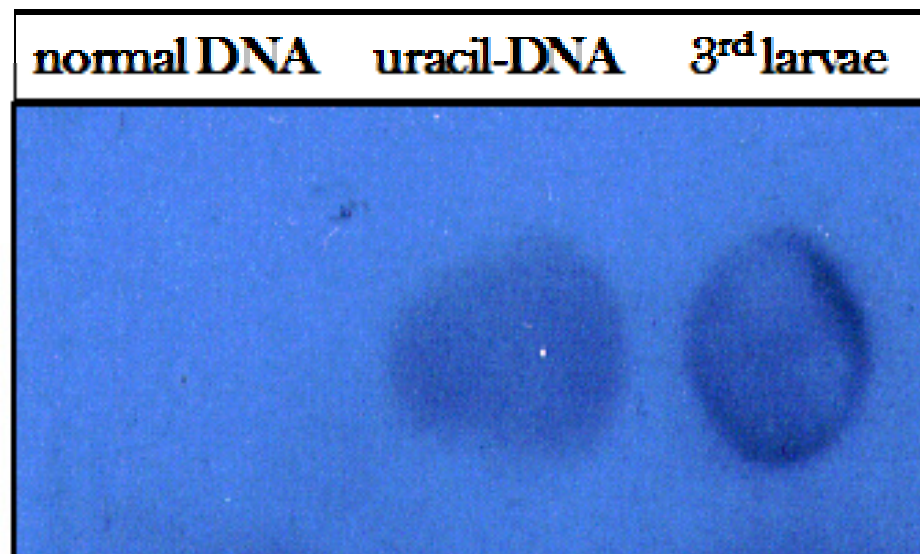

**Supplementary Figure S3**

Supplement: Figure S3 — Ung-ARP assay. UNG-ARP assay shows presence of uracil–DNA in Drosophila larvae. For negative and positive controls, genomic DNA samples from XL1 Blue and CJ236 ung-1, dut-1 E.coli strains were used respectively. CJ236 ung-1, dut-1 E.coli strain produces DNA with high uracil content (approx. 5500 uracil/million bases [8], [10]). (PDF) [file pgen.1002738.s003.pdf]

**A**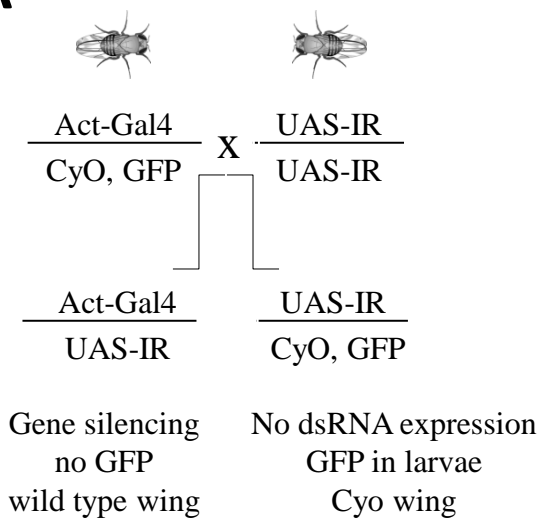**B**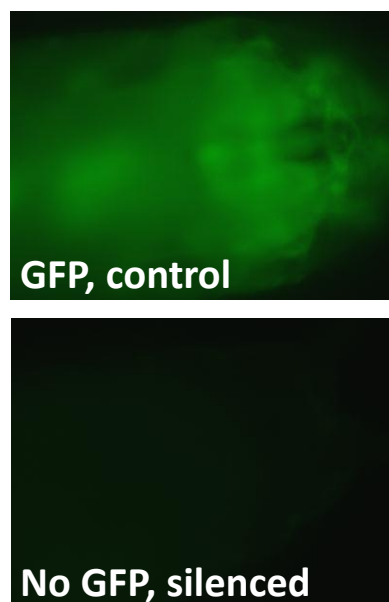**C**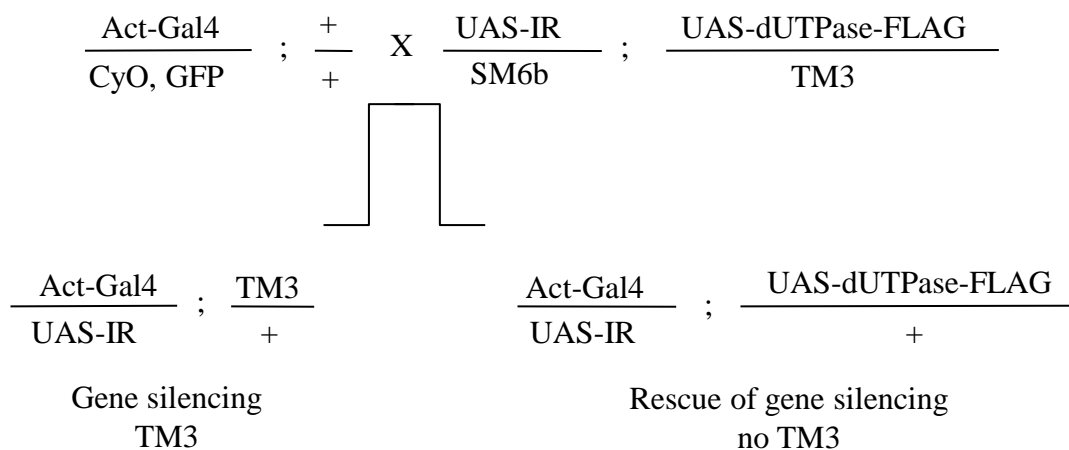**D**

Silencing allele

21883

21884

Rescue allele

-

DMdut20

DMdut29

-

DMdut20

DMdut29

dUTPase

tubulin

**Figure S4**

Supplement: Figure S4 — Scheme of crossing for silencing of dUTPase in Drosophila larvae and pupae and for rescue of dUTPase RNAi. Crossing schemes are shown on panel A and C: Act-Gal4 means Gal4 gene coupled with actin 5C promoter that result in ubiquitous and constitutive expression of yeast transcription factor, Gal4 in transgenic D. melanogaster driving transcription of silencing element (IR) following the UAS promoter. F1 generation has two genotypes: Act-Gal4/UAS-IR animals express dsRNA for dUTPase silencing, and have no markers; in UAS-IR/CyO, GFP animals, the silencing element is not activate, curly wing (CyO) and GFP markers expressed. Silenced and non-silenced animals are distinguishable at larvae/pupae and imago stages on the basis of GFP (panel B) and CyO markers, respectively. Crossing scheme for silencing is shown on panel C: UAS-dUTPase-FLAG stands for the rescue construct. Two relevant categories of the F1 generation can be unambiguously distinguished based on the phenotype of the marker mutations of the CyO, SM6b, and TM3 balancer chromosomes. The TM3 phenotype marks the gene silenced progenies, while the rescued animals show noTM3 phenotype. Panel D shows Western blot for dUTPase in silenced versus rescued animals. Note the absence of dUTPase protein in silenced animals (silencing alleles 21883 and 21884), whereas the presence of dUTPase proteins in the rescued animals (rescuing alleles DMDUT20 and DMDUT29). Equivalent total protein loading was verified by developing the blot also against tubulin using anti-Tubulin (E7, provided by M. Klymkowsky; Developmental Studies Hybridoma Bank, University of Iowa, Iowa city, IA). (PDF) [file pgen.1002738.s004.pdf]

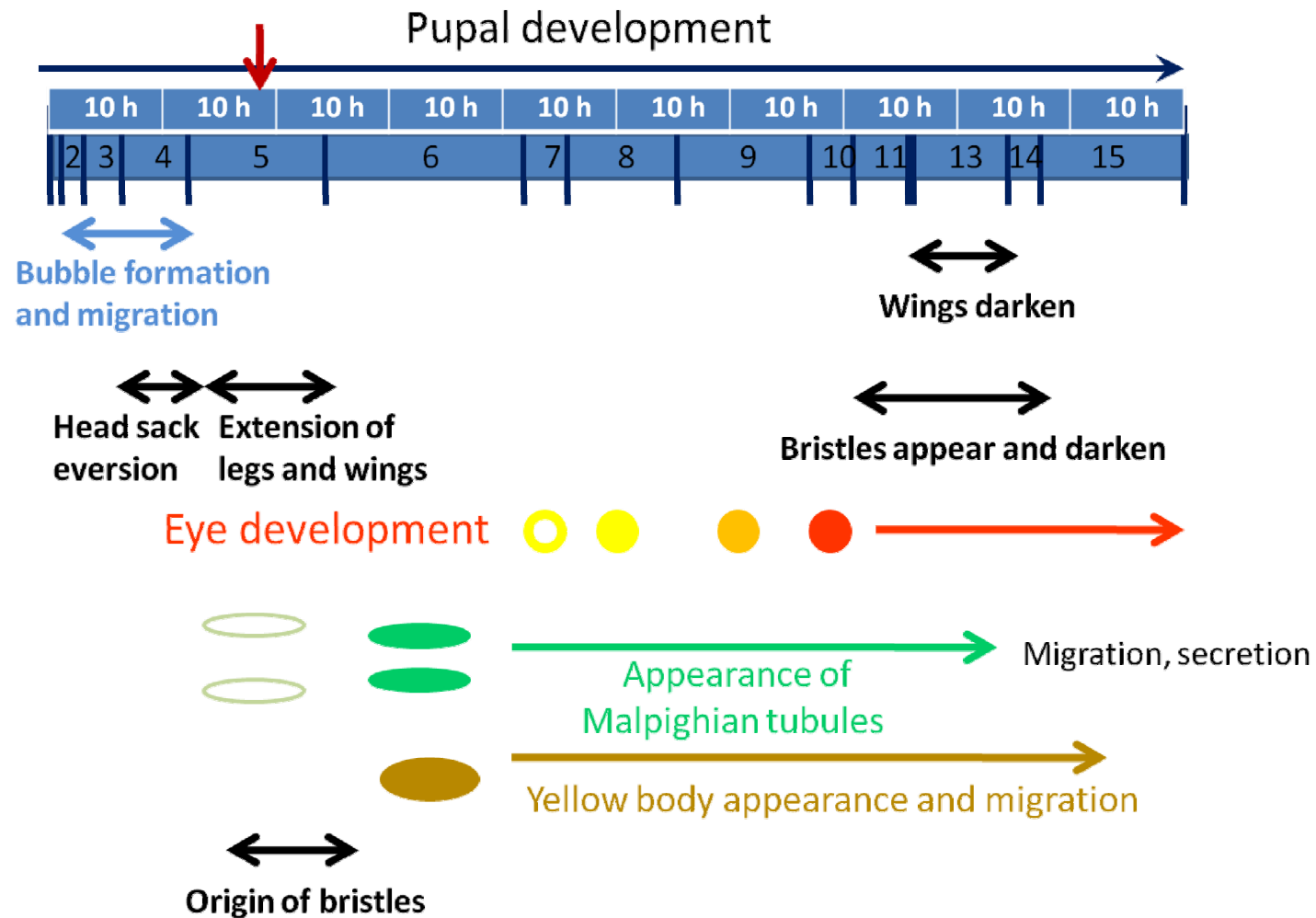

**Supplementary Figure S5**

Supplement: Figure S5 — Summary of pupal developmental processes. Red arrow shows the stage P5 (around 12–14 h after puparium formation) until lethality due to dUTPase silencing appear. (PDF) [file pgen.1002738.s005.pdf]

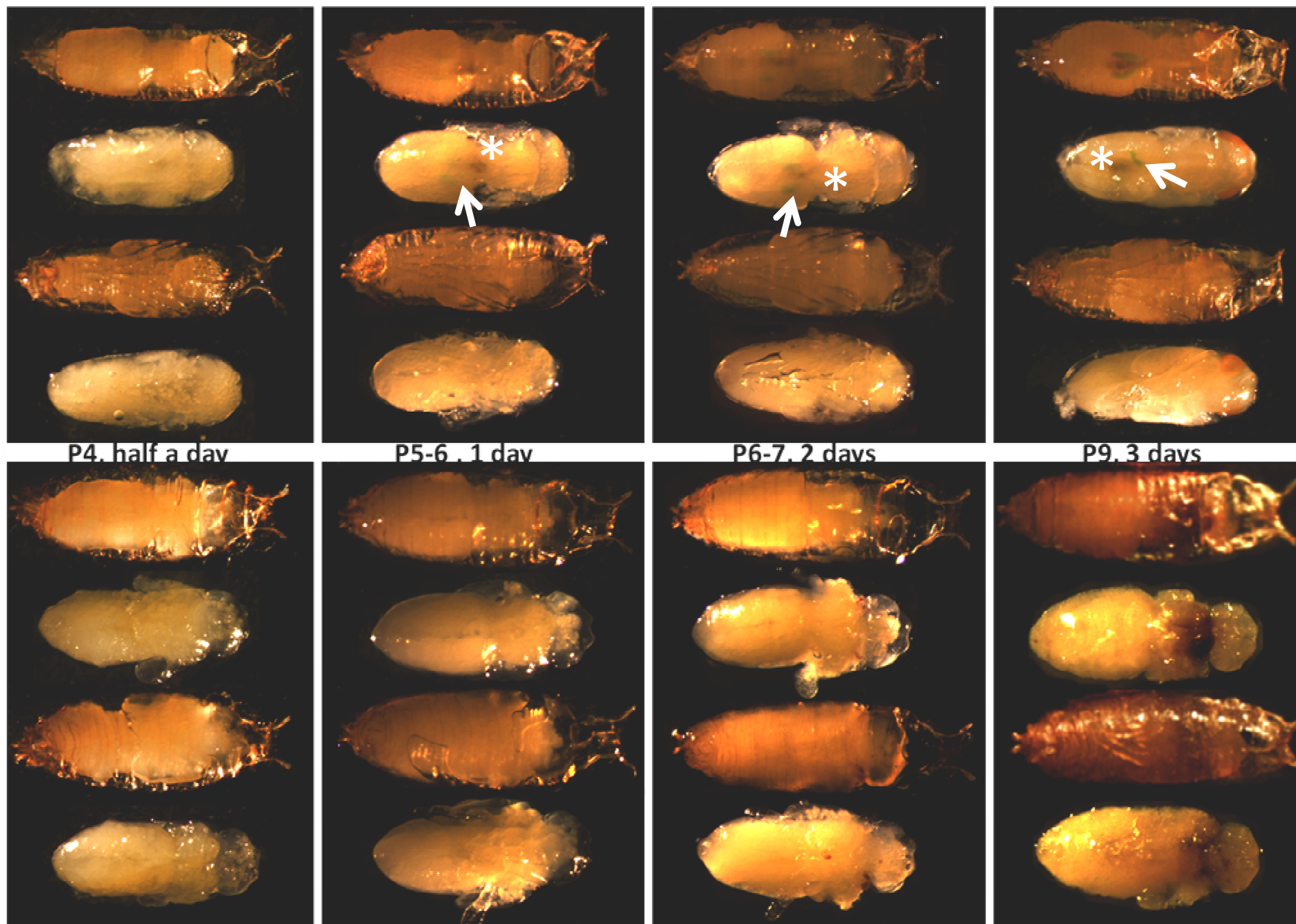

**Supplementary Figure S6**

Supplement: Figure S6 — Developmental arrest caused by dUTPase silencing in Drosophila pupae. Wild type (upper panels) and dUTPase silenced (bottom panels) pupae were compared in stages P4, P5–6, P6–7, and P9. Every panel shows four views of the same pupa: dorsal (upper two) and ventral (bottom two) with and without its puparium. Specific differences appear at or before P5: Malpighian tubules (arrows) and Yellow Body (asterices) never appears in dUTPase silenced pupae. (PDF) [file pgen.1002738.s006.pdf]

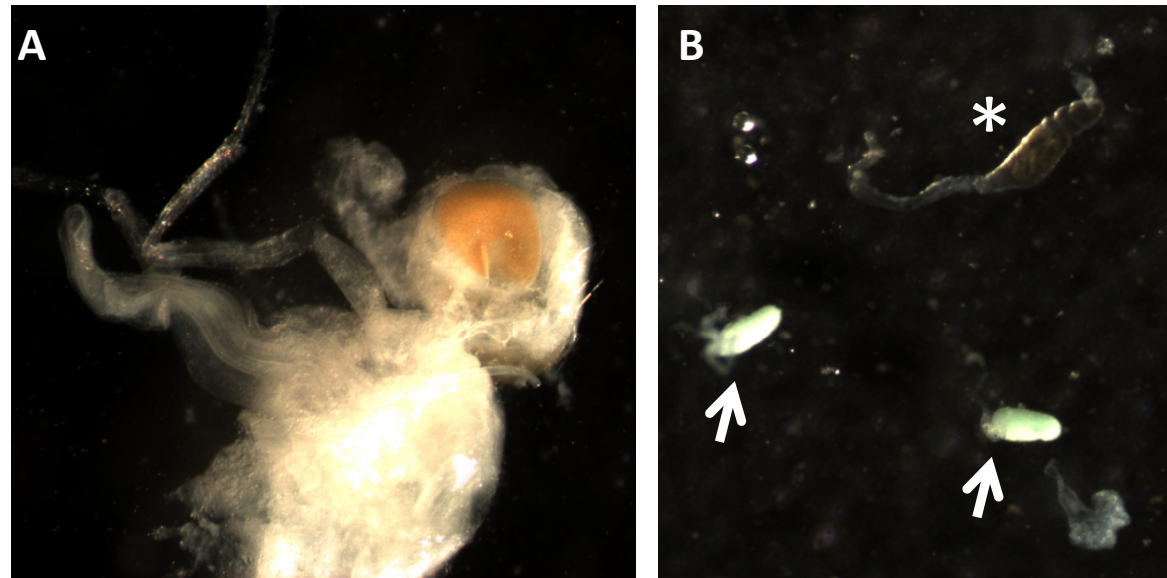

**Supplementary Figure S7**

Supplement: Figure S7 — Wild type structures of pharate adults 3 days after puparium formation. Wild type pupa was dissected at stage P11 where adult organs have already developed (A). Dissected Malpighian tubules (arrows on B) and Yellow Body (asterices on B) of wild type pupa these organs have never identified within dUTPase silenced pupae. (PDF) [file pgen.1002738.s007.pdf]

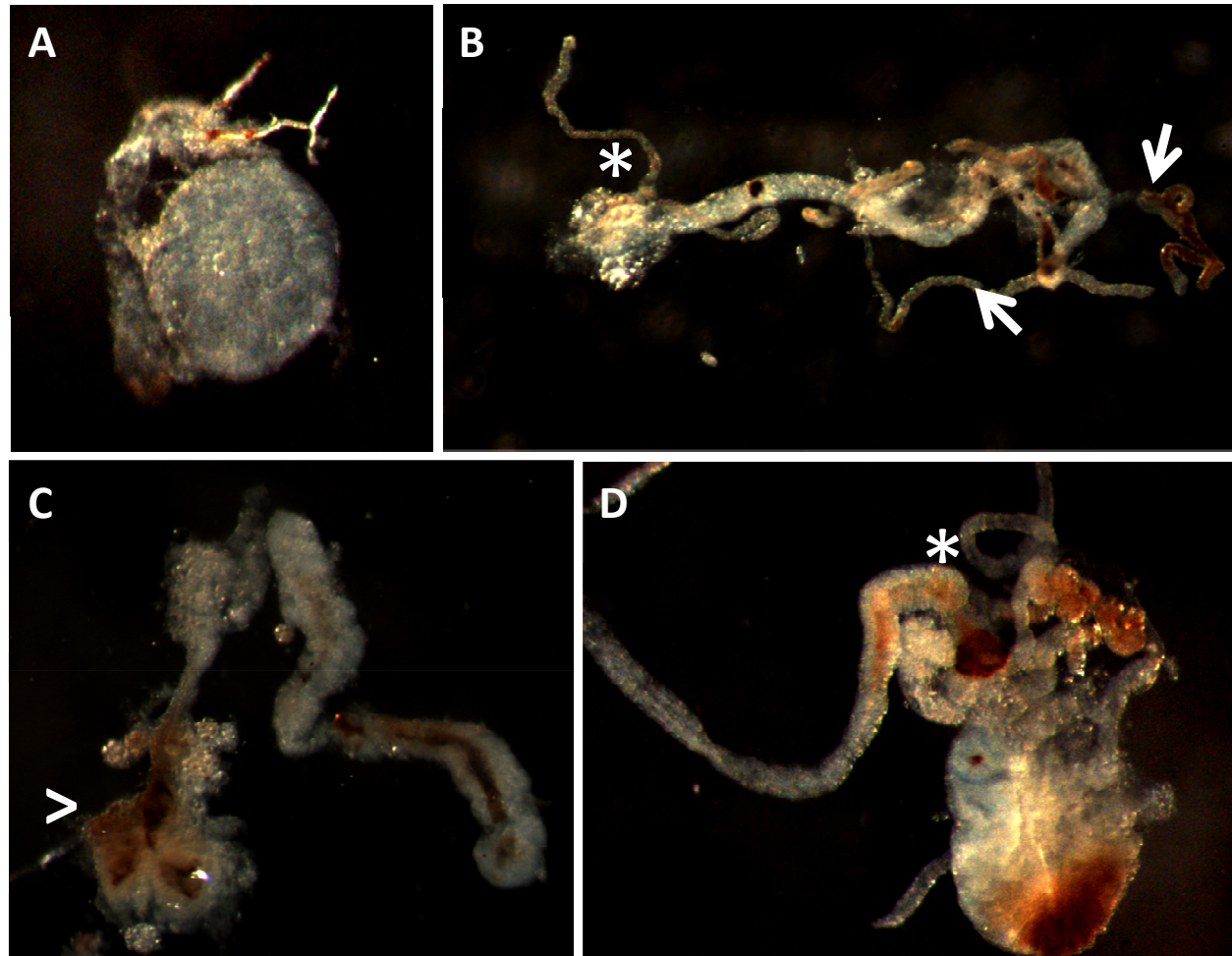

**Supplementary Figure S8**

Supplement: Figure S8 — Larval traits in dissected silenced pupae 3 days after puparium formation. Three days after puparium formation, dissected tissues of silenced pupae still preserve larval traits: testis is oval (A), foregut and gastric caeca show larval characteristics (B, D, asterices), Malpighian tubules (B, arrows) are thin characteristic for larval ones, and brain (C, white arrowhead) also preserves the basic structure of larval one. Darkened tissues may have resulted from necrosis, apoptosis or melanisation [36]. (PDF) [file pgen.1002738.s008.pdf]

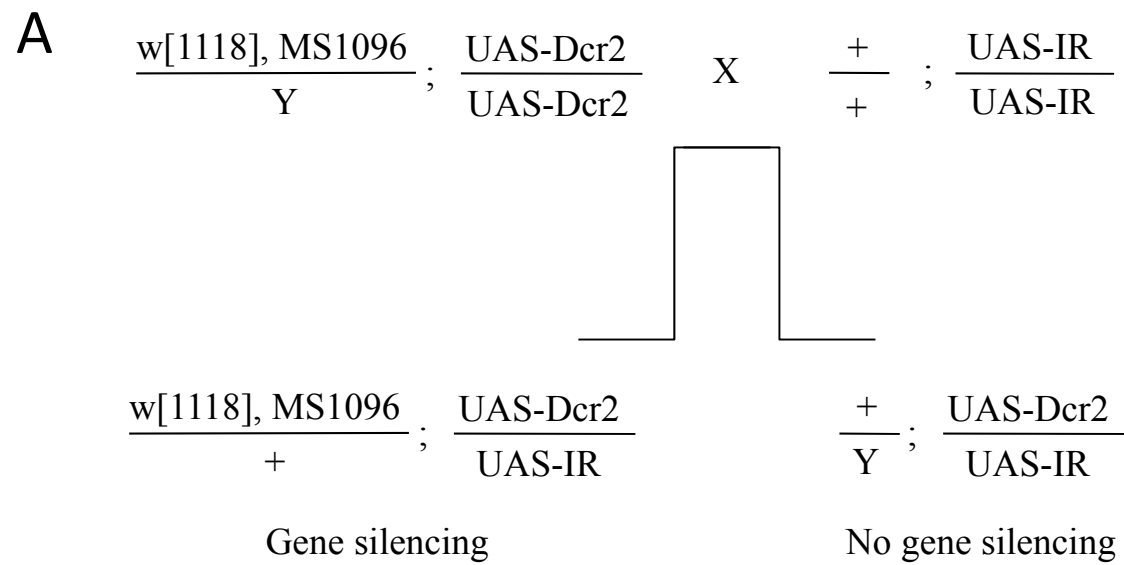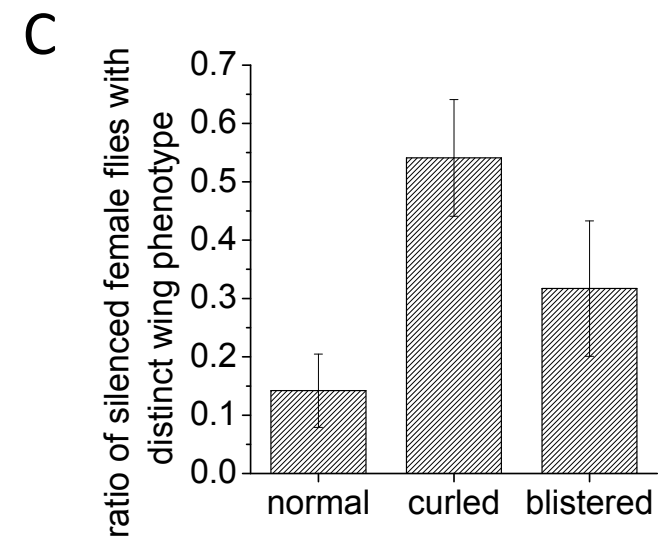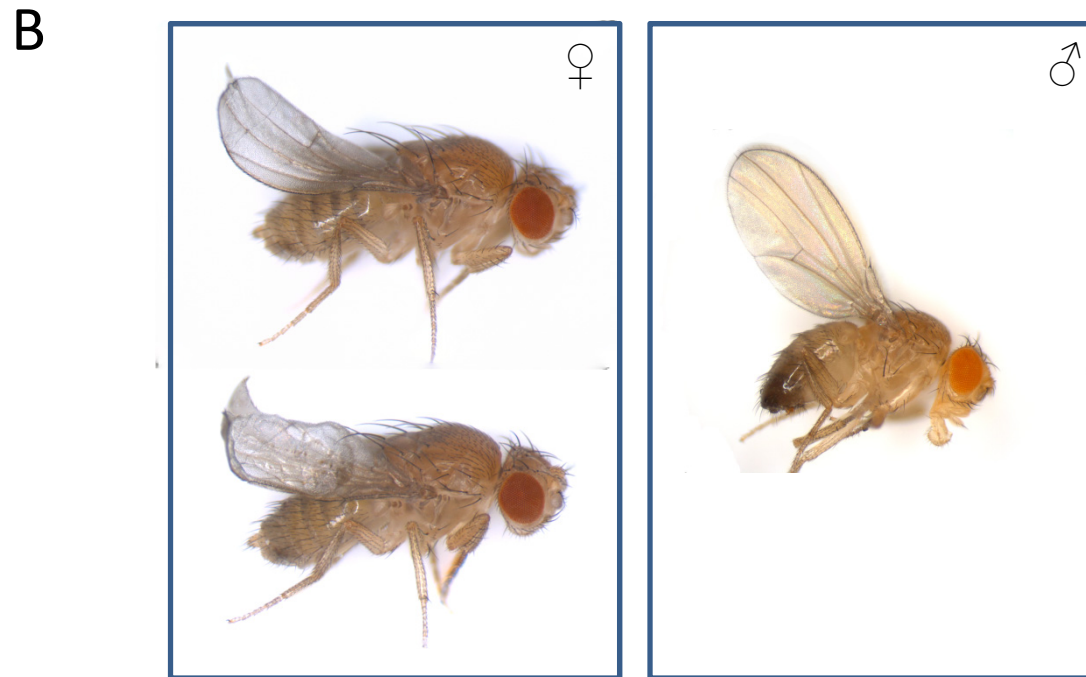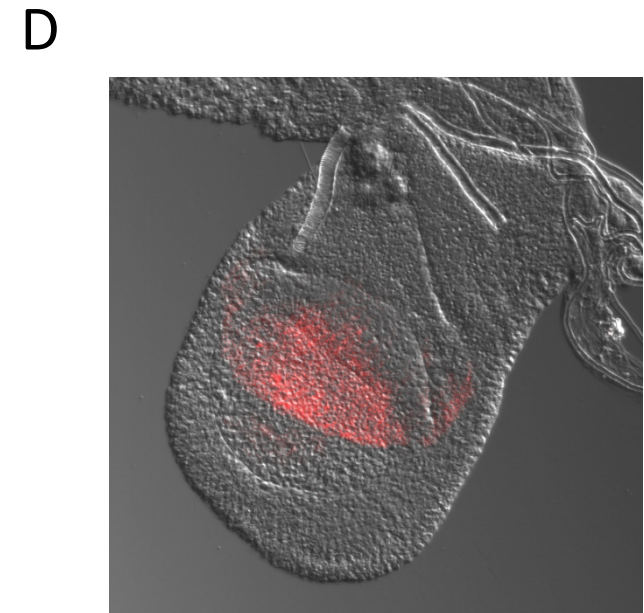

**Supplementary Figure S9**

Supplement: Figure S9 — Scheme of crossing for silencing of dUTPase in the dorsal compartment of Drosophila wing imaginal discs. Crossing scheme is shown on panel (A): virgin females of the MS1096 Gal4 enhancer trap line expressing Gal4 preferentially in the dorsal compartment of the wing and carrying UAS-Dicer2 in homozygous form on the second chromosome (Bloomington stock No. 25706) were crossed to males carrying the Gal4 inducible silencing element (UAS-IR) in homozygous form on the second chromosome. The silencing element was activated by the MS1096 driver [37] in female progenies only while F1 males served as an internal negative control where no silencing occurred. Silenced females exhibited dorsally curled wing phenotype (panel B) often with blisters. The penetrance of the phenotype was around 85%. About 35% of the silenced female progeny also showed blistering wings (panel C). Male progenies had no wing phenotype. Panel D shows the expression pattern of the MS1096 driver in the dorsal compartment of the wing disc visualized by crossing MS1096 females to UAS-MoesinCherry [51] males (panel D) (red fluorescent staining in the wing disc). MoesinCherry overexpressing female progeny had no wing phenotype. (PDF) [file pgen.1002738.s009.pdf]
