# Supplementary material for: Uracil-Containing DNA in Drosophila: Stability, Stage-Specific Accumulation, and Developmental Involvement
Source: PLoS Genet. 2012 Jun 7;8(6):e1002738. doi: 10.1371/journal.pgen.1002738 (PMC3369950; doi:10.1371/journal.pgen.1002738)
Supplement: Table S1 — Genomic position of UAS-IR constructs in dUTPase RNAi stocks. (PDF) [file pgen.1002738.s010.pdf]

Supplementary Tables

Table S1 Genomic position of UAS-IR constructs in dUTPase RNAi stocks

VDRC #	Insertion site	3' flanking gene	5' flanking gene
21883	2R, 60A, 19812632	CG5594	CG2812
21884	2L, 35A, 14234587	CG4551	-
