# Supplementary material for: Uracil-Containing DNA in Drosophila: Stability, Stage-Specific Accumulation, and Developmental Involvement
Source: PLoS Genet. 2012 Jun 7;8(6):e1002738. doi: 10.1371/journal.pgen.1002738 (PMC3369950; doi:10.1371/journal.pgen.1002738)
Supplement: Table S2 — dUTPase transgene rescues the dUTPase RNAi phenotype. Table shows the results of the rescue crosses. UAS-IR/SM6b; UAS-dUTPase-FLAG/TM3 males were crossed to Act-Gal4/CyO females (Figure S4). Two UAS-IR (21883 and 21884) and two transgenic rescue lines (DMDUT20 and DMDUT29) were combined. Number of progenies of the relevant F1 categories is shown. Gene silencing was complete since no UAS-IR/Act-GAl4; TM3/+ adult progeny was observed. However, when the dUTPase transgene was present, rescued animals survived to adulthood. (PDF) [file pgen.1002738.s011.pdf]

Table S2 dUTPase transgene rescues the dUTPase RNAi phenotype

Table shows the results of the rescue crosses. UAS-IR/SM6b; UAS-dUTPase-FLAG/TM3 males were crossed to Act-Gal4/CyO females (Fig. S4). Two UAS-IR (21883 and 21884) and two transgenic rescue lines (DmDUT20 and DMDUT29) were combined. Number of progenies of the relevant F1 categories is shown. Gene silencing was complete since no UAS-IR/Act-GAl4; TM3/+ adult progeny was observed. However, when the dUTPase transgene was present, rescued animals survived to adulthood.

		Gene silencing	Rescue of gene silencing
		UAS-IR/actGAl4;TM3/+	UAS-IR/actGal4;UAS dUTPase-FLAG /+
		number of F1 adult	
UAS-IR	actGal4;UAS dUTPase-FLAG		
VDRC #	transformant line ID		
21883	DMDUT20	0	72
21884	DMDUT20	0	68
21883	DMDUT29	0	29
21884	DMDUT29	0	70
