# Supplementary material for: Uracil-Containing DNA in Drosophila: Stability, Stage-Specific Accumulation, and Developmental Involvement
Source: PLoS Genet. 2012 Jun 7;8(6):e1002738. doi: 10.1371/journal.pgen.1002738 (PMC3369950; doi:10.1371/journal.pgen.1002738)
Supplement: Table S3 — Uracil–DNA repair is perturbed in Drosophila. Microarray data available on FlyBase were used. Table shows mRNA level for genes involved in different DNA repair pathways, elements of uracil–DNA repair are highlighted on grey background. ↓ indicates mRNA level decrease, ↑ mRNA level increase, ≈ no stage-specific change. Note that the overall base excision repair is down-regulated during larval development, but other DNA repair processes are not. (PDF) [file pgen.1002738.s012.pdf]

Table S3. Uracil-DNA repair is perturbed in *Drosophila*. Microarray data available on Flybase were used. Table shows mRNA level for genes involved in different DNA repair pathways, elements of uracil-DNA repair are highlighted on grey background. ↓ indicates mRNA level decrease, ↑ mRNA level increase, ≈ no stage-specific change. Note that the overall base excision repair is down-regulated during larval development, but other DNA repair processes are not.

pathway	enzyme	gene symbol	activity	larval expression compared to embryo	pupal expression compared to larva
Base Excision Repair	dUTPase	CG4584	prevention of uracil incorporation	↓	≈
	UNG	not encoded	UNG, main uracil-DNA glycosylase	-	-
	SMUG	CG5285	uracil-DNA glycosylase	↓	↑
	Thd1 (TDG)	CG1981	uracil-DNA glycosylase (U:G)	↓	↑
	NTHL1	CG9272	Oxidized pyrimidine base lesion	↓	↑
	Ogg1	CG1795	Oxidized purine base lesion	≈	≈
	Rrp1 (APEX1)	CG3178	AP endonuclease	↓	↑
	RpS3	CG6779	AP endonuclease	≈	↓
	RpLP0	CG7490	AP endonuclease	≈	↓
	XRCC1	CG4208	damaged DNA binding	↓	↑
	PARP	CG40411	DNA binding	↓	↑
	FEN1	CG8648	endonuclease activity	↑	≈
	ERCC5 (mus201)	CG10890	ss DNA endodeoxyribonuclease	↓	↑
Nucleo-tide Excision Repair	GEN	CG10670	XPG-like endonuclease	≈	↑
	XPA	CG6358	damaged DNA binding	≈	↑
Mismatch Repair	Mlh1	CG11482	mismatched DNA binding	↑	≈
	Pms2	CG8169	mismatched DNA binding	≈	↑
	Msh6	CG7003	mismatched DNA binding	≈	↑
	Msh2 (spel1)	CG4215	mismatched DNA binding	≈	↑
	PCNA (mus209)	CG9193	recruitment	↑	≈
DNA break	Ku70/Ku80	CG5247	DNA helicase	≈	≈
	Rad50	CG6339	nuclease	↑	↑
