# Supplementary material for: Uracil-Containing DNA in Drosophila: Stability, Stage-Specific Accumulation, and Developmental Involvement
Source: PLoS Genet. 2012 Jun 7;8(6):e1002738. doi: 10.1371/journal.pgen.1002738 (PMC3369950; doi:10.1371/journal.pgen.1002738)
Supplement: Text S1 — Supplementary information. Includes Supplementary Materials and Methods and Supplementary References. (DOC) [file pgen.1002738.s013.doc]

**Text S1**

Supplementary information for:

**Uracil-containing DNA in *Drosophila*: Stability, Stage-specific Accumulation, and Developmental Involvement**

Villő Muha^1#^, András Horváth^1#^, Angéla Békési^1^, Mária Pukáncsik^1^, Barbara Hodoscsek^1^, Gábor Merényi^1^, Gergely Róna^1^, Júlia Batki^1^, István Kiss^2^, Ferenc Jankovics^2^, Péter Vilmos^2^, Miklós Erdélyi^2^ & Beáta G. Vértessy^1,3^

*^1^Institute of Enzymology, Research Center for Natural Sciences, Hungarian Academy of Science, Budapest, Hungary*

*^2^Institute of Genetics, BRC, Hungarian Academy of Science, Szeged, Hungary*

*^3^Department of Applied Biotechnology and Food Science, University of Technology and Economics, Budapest, Hungary*

**^#^ Joint first authors**

*Table of Contents:*

**Supplementary Materials and Methods** (pages 2-4)

**Supplementary References** (page 5)

**Supplementary Figure Legends** (pages 6-9)

**Supplementary Table Legends** (page 10)

**Supplementary Materials and Methods**

**Real-time PCR for quantification of dUTPase mRNA level**

Total RNA from *Drosophila* samples were prepared (RNeasy Plus Mini Kit, QIAGEN). RNA (1 μg) was reverse-transcribed with (dT)_18_ primers (Fermentas) and M-MuLV Reverse Transcriptase (NEB) according to the manufacturer’s protocol. cDNA was measured by Quantitative PCR with EvaGreen (Bioline) and Immomix PCR master mix (Bioline) on Rotor-Gene 3000 instrument (Corbett Life Science). Rp49, an endogenous reference gene was used. Non-reverse-transcribed control samples confirmed that the RNA sample was DNA-free. Relative expression ratios of the examined genes were calculated using the comparative Ct method (ΔΔCt). For dUTPase 5'-GAGCGTATCTTCTATCCGCAAC-3' and 5'-GCGGCAATAGTTCCTCGTTTAC-3'; for Rp49 5’-ATACAGGCCCAAGATCGTGAAG-3’ and 5’-GCACCAGGAACTTCTTGAATCC-3’ primers were used.

**Details of** dUTPase silencing by RNA interference and rescue of RNAi using the ActGal4 driver

Insertion site of the P-element containing the UAS-IR sequence was determined by inverse PCR and sequencing according to BDGP protocol. Results are summarized in Table S1. Overall silencing was induced by crossing UAS-IR homozygous males to virgin females carrying ActGal4/CyO, GFP drivers (Figure S4 A-B) and vice versa. Progeny were raised at 25°C and scored according to GFP marker in larvae or CyO wing marker in imago. Animals lacking GFP and CyO wing markers express the IR construct thus silence dUTPase. Efficiency of silencing was determined by performing Western blot with anti-dUTPase polyclonal antibody as described previously .

For rescue of this silencing, we followed the procedure described below. PCR was applied on Drosophila genomic DNA by the primers DmDut_attB1Fw (5’-GGG GAC AAG TTT GTA CAA AAA AGC AGG CTC CGC CAC CAT GCC ATC AAC CGA TTT CG-3’) and DmDut_attB2Rev (5’-GGG GAC CAC TTT GTA CAA GAA AGC TGG GTT CGT AGC AAC AGG AGC CGG-3’) . The primers were designed to amplify the nuclear isoform of Drosophila dUTPase. After purification, fragment was recombined into pDONR221 entry vector by Gateway BP Clonase mix (Invitrogen). Resulting plasmid was then recombined into pTWF vector (DGRC) encoding P1 and P2 element transposition sites and a FLAG C-terminal fusion tag by Gateway LR Clonase mix (Invitrogen). Successful recombination was tested by sequencing. Using this plasmid, strains were generated by normal P-element transformation protocol. Two independent transformant lines were generated (DMDUT20 and DMDUT29). Both transformant lines were UAS-IR/SM6b;UAS-dUTPase-FLAG/TM3. Silencing was rescued by crossing UAS-IR/SM6b;UAS-dUTPase-FLAG/TM3 males to Act-Gal4/CyO females (Figure S4C).

Progeny were raised at 25°C and scored according to the markers of the CyO, SM6b, and TM3 balancer chromosomes. Results are reported in Table S2 and indicate full rescue of dUTPase RNAi by the co-expression of the dUTPase transgene.

**Silencing of dUTPase using the MS1096 driver**

Males carrying the MS1096 driver (Bloomington Stock No. 25706) driver on their X- chromosome were crossed with virgin homozygous UAS-IR or UAS-MoesinCherry females. Females from progeny carried the MS1096 driver, the UAS-Dicer 2 and UAS-IR or UAS-MoesinCherry alleles, while males carried only the UAS-Dicer 2 and UAS-IR or UAS-MoesinCherry alleles (Figure S9A). Progeny were raised at 25°C. Driver efficiency and specificity was tested on female progeny carrying the MoesinCherry gene by imaging the fluorescence of wing discs (Figure S9D).

**UNG- Aldehyde Reactive Probe assay**

Aldehyde reactive probe (ARP) assay was performed using ARP reagent (Dojindo Molecular Technologies). Reaction mixtures contained 0,1 mg/ml *E.coli* plasmid DNA or *Drosophila* larval genomic DNA in 25 mM Tris-HCl (pH=7.5), 1 mM MgCl_2_, 0.1 mg/ml BSA, 2 mM ARP assay buffer and 10^-2^ U/ml UNG protein (Sigma). Reaction mixtures were incubated for 60 min at 37 ºC, spotted on nitrocellulose membrane and analyzed as described in . Biotin-tagged AP sites were detected using streptavidin-conjugated horseradish peroxidase (Sigma).

**Staging of Drosophila pupae**

To determine the time interval necessary for puparium formation, crossed flies were allowed to deposit eggs for 2 hours per tubes. From these synchronous tubes, white pupae were collected every 3-4 hours, displaced onto plates with hard-agar medium, and the time required to reach this stage was recorded. On the plates, their further development was followed, and their genotypes were determined based on GFP, and curly wing markers. Cumulative data of the numbers of animals having undergone puparium formation in certain time points are shown in Figure 3B.

To stage the silenced and non-silenced pupae, wandering larvae were collected from the wall of the tubes. GFP-expression was determined on living animals using a Leica DMLS fluorescence microscope. Silenced and non-silenced control animals were isolated based on their GFP expression. Synchronous *Drosophila* pupae were collected by checking these tubes for white prepupae every 3-4 hours, displacing them onto plates with hard-agar medium. The actual pupal stages were determined based on the observable markers of wild type pupae as described in (Figure S5, ).

**Supplemementary References**

1. Bekesi A, Zagyva I, Hunyadi-Gulyas E, Pongracz V, Kovari J, et al. (2004) Developmental Regulation of dUTPase in Drosophila melanogaster. J Biol Chem 279: 22362-22370.

2. Capdevila J, Guerrero I (1994) Targeted expression of the signaling molecule decapentaplegic induces pattern duplications and growth alterations in Drosophila wings. EMBO J 13: 4459-4468.

3. Millard TH, Martin P (2008) Dynamic analysis of filopodial interactions during the zippering phase of Drosophila dorsal closure. Development 135: 621-626.

4. Lari SU, Chen CY, Vertessy BG, Morre J, Bennett SE (2006) Quantitative determination of uracil residues in Escherichia coli DNA: Contribution of ung, dug, and dut genes to uracil avoidance. DNA Repair (Amst) 5: 1407-1420.

5. Bainbridge SP, Bownes M (1981) Staging the metamorphosis of Drosophila melanogaster. J Embryol Exp Morphol 66: 57-80.

6. Horvath A, Vertessy BG (2010) A one-step method for quantitative determination of uracil in DNA by real-time PCR. Nucleic Acids Res 38: e196.

7. Minakhina S, Steward R (2006) Melanotic mutants in Drosophila: pathways and phenotypes. Genetics 174: 253-263.

**Supplementary Figure Legends**

**Figure S1. Percentage of fluorescent cells upon transfection with normal (T pl.) or uracil-substituted plasmids (U pl.). A** *Drosophila* S2 cells, **B** HeLa cells. The number of observed fluorescent cells is also presented within the bars together with the total number of scored cells (shown in brackets).

**Figure S2. Genomic uracil content of embryo is under detection limit.** Uracil content of *Drosophila* embryonic genome compared to that of DNA plasmid purified from wild-type *E. coli*. Both of the samples showed a value under the detection limit.

**Figure S3. Ung-ARP assay.** UNG-ARP assay shows presence of uracil-DNA in *Drosophila* larvae. For negative and positive controls, genomic DNA samples from XL1 Blue and CJ236 *ung-1, dut-1* *E.coli* strains were used respectively. CJ236 *ung-1, dut-1* *E.coli* strain produces DNA with high uracil content (approx. 5500 uracil / million bases ).

**Figure S4. Scheme of crossing for silencing of dUTPase in *Drosophila* larvae and pupae and for rescue of dUTPase RNAi.** Crossing schemes are shown on panel **A** and **C**: Act-Gal4 means Gal4 gene coupled with actin 5C promoter that result in ubiquitous and constitutive expression of yeast transcription factor, Gal4 in transgenic *D. melanogaster* driving transcription of silencing element (IR) following the UAS promoter. F1 generation has two genotypes: Act-Gal4/UAS-IR animals express dsRNA for dUTPase silencing, and have no markers; in UAS-IR/CyO, GFP animals, the silencing element is not activate, curly wing (CyO) and GFP markers expressed. Silenced and non-silenced animals are distinguishable at larvae/pupae and imago stages on the basis of GFP (panel **B**) and CyO markers, respectively.

Crossing scheme for silencing is shown on panel **C**: UAS-dUTPase-FLAG stands for the rescue construct. Two relevant categories of the F1 generation can be unambiguously distinguished based on the phenotype of the marker mutations of the CyO, SM6b, and TM3 balancer chromosomes. The TM3 phenotype marks the gene silenced progenies, while the rescued animals show noTM3 phenotype.

Panel **D** shows Western blot for dUTPase in silenced vs. rescued animals. Note the absence of dUTPase protein in silenced animals (silencing alleles 21883 and 21884), whereas the presence of dUTPase proteins in the rescued animals (rescuing alleles DMDUT20 and DMDUR29). Equivalent total protein loading was verified by developing the blot also against tubulin using anti-Tubulin (E7, provided by M. Klymkowsky; Developmental Studies Hybridoma Bank, University of Iowa, Iowa city, IA).

**Figure S5**. **Summary of pupal developmental processes.** Red arrow shows the stage P5 (around 12-14h after puparium formation) until lethality due to dUTPase silencing appear.

**Figure S6. Developmental arrest caused by dUTPase silencing in *Drosophila* pupae.** Wild type (upper panels) and dUTPase silenced (bottom panels) pupae were compared in stages P4, P5-6, P6-7, and P9. Every panel shows four views of the same pupa: dorsal (upper two) and ventral (bottom two) with and without its puparium. Specific differences appear at or before P5: Malpighian tubules (arrows) and Yellow Body (asterices) never appears in dUTPase silenced pupae.

**Figure S7**. **Wild type structures of pharate adults 3 days after puparium formation.** Wild type pupa was dissected at stage P11 where adult organs have already developed (A). Dissected Malpighian tubules (arrows on B) and Yellow Body (asterices on B) of wild type pupa these organs have never identified within dUTPase silenced pupae.

**Figure S8**. **Larval traits in dissected silenced pupae 3 days after puparium formation.** Three days after puparium formation, dissected tissues of silenced pupae still preserve larval traits: testis is oval (A), foregut and gastric caeca show larval characteristics (B, D, asterices), Malpighian tubules (B, arrows) are thin characteristic for larval ones, and brain (C, white arrowhead) also preserves the basic structure of larval one. Darkened tissues may have resulted from necrosis, apoptosis or melanisation .

**Figure S9.** **Scheme of crossing for silencing of dUTPase in the dorsal compartment of *Drosophila* wing imaginal discs.** Crossing scheme is shown on panel (**A)**: virgin females of the MS1096 Gal4 enhancer trap line expressing Gal4 preferentially in the dorsal compartment of the wing and carrying UAS-Dicer2 in homozygous form on the second chromosome (Bloomington stock No. 25706) were crossed to males carrying the Gal4 inducible silencing element (UAS-IR) in homozygous form on the second chromosome. The silencing element was activated by the MS1096 driver in female progenies only while F1 males served as an internal negative control where no silencing occurred. Silenced females exhibited dorsally curled wing phenotype (panel **B**) often with blisters. The penetrance of the phenotype was around 85%. About 35% of the silenced female progeny also showed blistering wings (panel **C**). Male progenies had no wing phenotype. Panel **D** shows the expression pattern of the MS1096 driver in the dorsal compartment of the wing disc visualized by crossing MS1096 females to UAS-MoesinCherry males (panel **D**) (red fluorescent staining in the wing disc). MoesinCherry overexpressing female progeny had no wing phenotype.

**Supplementary Table Legends**

**Table S1 Genomic position of UAS-IR constructs in dUTPase RNAi stocks**

**Table S2 dUTPase transgene rescues the dUTPase RNAi phenotype.** Table shows the results of the rescue crosses. UAS-IR/SM6b; UAS-dUTPase-FLAG/TM3 males were crossed to Act-Gal4/CyO females (Figure S4). Two UAS-IR (21883 and 21884) and two transgenic rescue lines (DMDUT20 and DMDUT29) were combined. Number of progenies of the relevant F1 categories is shown. Gene silencing was complete since no UAS-IR/Act-GAl4; TM3/+ adult progeny was observed. However, when the dUTPase transgene was present, rescued animals survived to adulthood.

**Table S3. Uracil-DNA repair is perturbed in *Drosophila*.** Microarray data available on FlyBase were used. Table shows mRNA level for genes involved in different DNA repair pathways, elements of uracil-DNA repair are highlighted on grey background. ↓ indicates mRNA level decrease, ↑ mRNA level increase, ≈ no stage-specific change. Note that the overall base excision repair is down-regulated during larval development, but other DNA repair processes are not.
